# Investigation of the Mechanical, Fatigue, and Creep Properties of PA6/GO Nanocomposites Manufactured by a Combination of Melt and Solvent Mixing

**DOI:** 10.3390/polym17091186

**Published:** 2025-04-27

**Authors:** Mehmet Palabiyik, Serhat Aydin, Oguzkan Senturk

**Affiliations:** 1 Faculty of Mechanical Engineering, Istanbul Technical University, Istanbul 34437, Turkey; serhat.aydin@gevernova.com (S.A.); oguzkan.senturk@gevernova.com (O.S.); 2 GE Vernova Grid Solutions, Gebze 41410, Turkey

**Keywords:** creep, fatigue, GO, mechanical, nanomaterials, PA6, polymers and plastics

## Abstract

This study investigated the mechanical, fatigue, and creep properties of polyamide 6 (PA6)/graphene oxide (GO) nanocomposites manufactured by a combination of melt and solvent mixing. Results showed that increasing GO content improved tensile and bending properties and reduced temperature dependence. The tensile modulus and strength of PA6/GO nanocomposite containing 1 wt.% GO (PA6 + 1GO) were measured with an increment of 33% and 37%, respectively, compared with neat PA6. The reduction in tensile strength occurred gradually with the increasing amount of GO. As the temperature increased from 25 °C to 70 °C, the tensile strength of PA6 and PA6 + 1GO decreased by 20% and 4%, respectively. Fatigue tests demonstrated that the rigid GO particles hindered the deformation capability of the matrix and facilitated crack propagation. While the PA6 reached 10^5^ cycles at 60% of its tensile strength, PA6 + 1GO was able to reach 10^5^ cycles at 35% of its tensile strength. Dynamic mechanical analysis (DMA) revealed that GO enhanced both storage modulus and glass transition temperature (T_g_). Creep tests demonstrated better deformation resistance under stress in PA6/GO nanocomposites compared to pure PA6. After a 10 h creep test, the decrease in creep strain was observed as 52.4% for PA6 + 1GO.

## 1. Introduction

Novel innovations in polymers contribute to future technological progress and bring about changes in engineering applications. The development of next-generation engineering polymers is crucial not only for industrial uses but also for providing environmental and biological solutions, promoting sustainability. Therefore, research and development in polymer science are highly valuable both scientifically and industrially. Among engineering polymers, polyamide 6 (PA6) is distinguished by its low production cost, simpler processing, and high melting temperature, making it one of the most used polymers [1]. Many industrial applications use polymer nanocomposites because of their unique combination of properties that polymers, ceramic materials, and metals lack [2,3,4]. The key components that affect the electrical, chemical, optical, and mechanical characteristics of nanocomposites are nanoscale organic and inorganic materials.

Carbon nanotubes, fullerenes, etc., have become the preferred nanomaterials in polymer composites due to their high mechanical properties [5,6,7,8,9,10,11]. Graphene (G), the stiffest material ever measured, is known for its high mechanical strength, excellent electrical conductivity, and low weight [12]. Its oxidized form, GO, stands out with properties like solubility in water and surface reactivity [13,14]. One of the key problems encountered in nano-filled polymer composites is the non-homogeneous dispersion of GO within the polymer matrix and the lack of homogeneous interface interactions [15,16,17]. Melt mixing is inadequate for the separation and dispersion of agglomerated graphene oxide layers inside a polymer matrix, since the viscous forces during the extrusion process are insufficient to separate graphene oxides with high surface energy that are linked by strong Van der Waals forces. Currently, methods such as melt mixing, solvent mixing, surface enhancement using surfactants, and in situ polymerization emerge as solutions to ensure homogeneous dispersion of GO within the polymer matrix [18,19,20]. To address the issues of agglomeration and non-homogeneous dispersion in polymer nanocomposites, a combination of melt and solvent mixing presents an alternative and effective solution [21,22]. Additionally, the properties of composites, particularly those reinforced with rigid particles such as GO, are heavily influenced by the interfacial interactions between the filler and the polymer matrix. The efficiency of the reinforcement effect is contingent on the quality of these interactions. Specifically, the graphene–matrix interface plays a pivotal role in determining the overall performance of the nanocomposite. Homogeneously dispersed GO particles, coupled with strong interfacial bonding, can significantly enhance the mechanical, thermal, and creep properties of PA6 [23,24]. The interaction between GO and the polymer matrix is a critical factor in determining the material’s overall properties [25].

Researchers have investigated the impact of GO on the characteristics of polymers. Moreover, the impacts of other organic and inorganic nanomaterials on polymers have also been widely investigated [26,27,28,29]. Maio et al. [30] aimed to combine melt and solvent mixing to prevent agglomeration problems of GO in the melt mixing process within the PA6 and EVA matrix. Characterization tests showed that the melt mixing was insufficient for the homogeneous distribution of GO in the polymer matrix. In mechanical tests performed after integrating the two methods, significant increases were observed in tensile and bending strengths. Khan et al. [31] studied the enhancement of the mechanical and thermal properties of polyurethane composites by adding G exfoliated with a solvent. Mechanical tests using different G ratios showed that the addition of G increased the tensile strength by 30%, with the composite containing 2% G achieving a tensile strength of 60 MPa. Impact strength showed significant improvement, reaching 8 kJ/m^2^ with 1% G compared to the control group. Głuchowski et al. [32] reported that as GO content increased in PA6, the elastic modulus rose by 18%, with a tensile strength of 77.5 MPa for the 1% GO composite. The 0.5% and 1% GO composites showed the greatest improvement, reaching 3020 MPa in elastic modulus, compared to 2560 MPa for pure PA6. Senturk et al. [33] examined the mechanical, viscoelastic, and tribological properties of PA6/HDPE + GO polymer blend nanocomposites manufactured by melt and solvent mixing. The increase in GO content improved tensile and bending properties, but reduced impact resistance. As the GO content increased, the storage modulus rose, Tan Delta diminished, and the T_g_ elevated. Xu et al. [34] evaluated the mechanical properties of PA6-nano-SiO_2_ nanocomposites manufactured by a combination of in situ polymerization and melt mixing methods. The results showed that the addition of nano-SiO_2_ to the PA6 matrix improved the mechanical properties. Scaffaro et al. [35] highlighted the interfacial interaction between GO and the polymer matrix. Significant improvements were noted in the mechanical properties of PA6 when GO was incorporated, particularly in tensile strength and modulus. Similarly, Ambhorkar et al. [36] reported that nanofillers significantly improved thermal stability and creep resistance in polymer matrices.

In this study, the focus was on ensuring the effective transfer of the superior properties of GO during the manufacturing of nanocomposites and analyzing the influence of GO content on the results. To this end, the mechanical, fatigue, and creep properties of PA6/GO nanocomposites obtained by combining melt and solvent mixing were investigated. No study was found in the literature that addresses the fatigue and creep properties of PA6/GO nanocomposites. However, there is a limited number of publications on the mechanical and viscoelastic properties of GO-based PA6 nanocomposites.

Tensile tests at different temperatures, together with bending tests and notched impact tests, were performed to assess the mechanical properties. The main reason for conducting tensile testing at varying temperatures is to simulate actual conditions in which materials experience thermal fluctuations. This is especially important for applications in areas where materials often undergo varying temperatures, and their performance under such conditions is essential. Tensile testing at various temperatures helps to evaluate material behavior, particularly near or above T_g_. After the tensile tests, the morphology of the fracture surfaces was analyzed using SEM, and the existence of nonhomogeneous dispersion and aggregation, a significant problem in utilizing GO or other nanofiller elements inside the polymer matrix, was investigated. Fatigue tests were performed to monitor the influence of GO on the strength of nanocomposites subjected to cycling loading. To define the viscoelastic properties, DMA tests were conducted. Tensile and creep test temperatures were established based on T_g_, as obtained from DMA. The creep tests were used to evaluate and measure the steady-state creep by the constant stress and temperature effect of increasing GO content. Creep tests performed above T_g_ help assess how GO affects the deformation of PA6/GO nanocomposites over time when a steady load is applied at elevated temperature. In the nanocomposites, issues such as non-homogeneous distribution and agglomeration, which are commonly encountered when using GO or other nanofiller materials in a polymer matrix, were discussed, and how these issues can be addressed through the chosen manufacturing method was assessed.

## 2. Materials and Methods

### 2.1. Materials

Polymer nanocomposites were prepared using PA6 and GO in four different formulations. Domo Domamid 6AV PA6, Leuna, Germany was used in the fabrication of polymer nanocomposites. During the preparation of the PA6/GO nanocomposites, GO nanopowder was obtained from Hazerfen Graphene, Kocaeli, Turkey. Ethanol and formic acid of analytical grade were purchased from Sigma-Aldrich, Darmstadt, Germany. Material properties of PA6 and GO are given in Table 1.

### 2.2. Manufacturing of Polymer Nanocomposites

**Figure 1:**
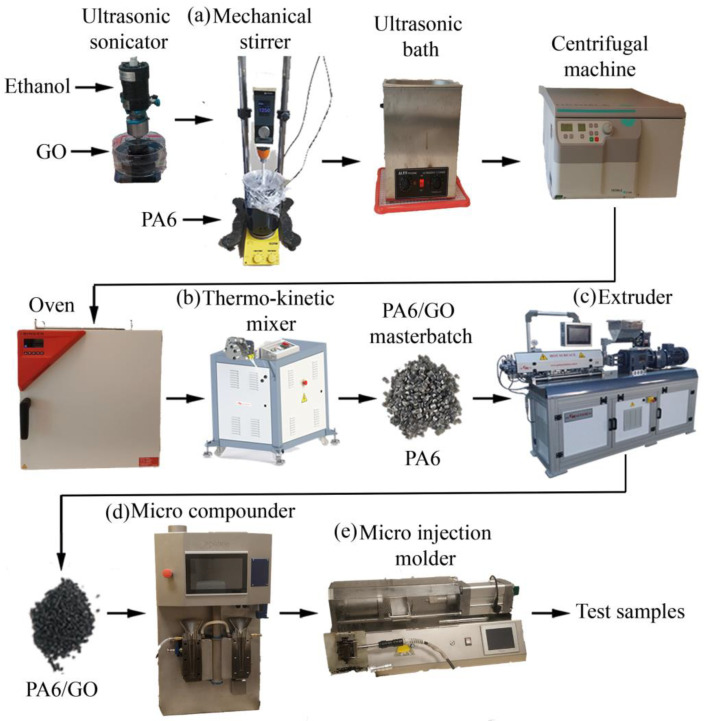
Schematic representation of the manufacturing process of the PA6/GO nanocomposites.

During the manufacturing process of the PA6/GO nanocomposites, a masterbatch containing 2% weight fraction of GO was initially prepared. Briefly, 1.25 kg of PA6 and 3 L of formic acid were added into a temperature-controlled mechanical stirrer (Figure 1a). The temperature was set to 100 °C, and the speed of the mechanical stirrer was maintained at 1250 rpm throughout the process. The stirring continued for 24 h to ensure that the PA6 was completely dissolved in the formic acid. Then, 250 mL of ethanol and 25 g of GO mixture were subjected to ultrasonic treatment for 100 min to disperse the agglomerated GO particles. The obtained GO/ethanol mixture was slowly added to the PA6/formic acid solution and stirred for 48 h. The resulting mixture was transferred into a container and positioned in a fume hood to dry and harden for 2 days. The hardened mixture was ground using a mechanical grinder. To separate the PA6/GO mixture from the formic acid, a washing process was carried out using ethanol. The PA6/GO mixture containing ethanol was then left in an ultrasonic bath for 2 h, followed by centrifugation at 8000 rpm for 5 min to separate the components. The washing procedure utilizing ethanol persisted till the total elimination of formic acid was achieved. Subsequently, the final mixture was subjected to a drying process at 60 °C in an oven. The dried mixture was then ground again using a mechanical grinder. The ground PA6/GO mixture was further dried at 85 °C for 24 h to remove moisture. The resulting mixture was immediately loaded into a thermo-kinetic mixer (Figure 1b). The mixture was then subjected to cold pressing to form a sheet. The sheet of PA6/GO underwent a granulation process using a knife mill. The drying process was repeated, and the materials that had been effectively prepared and dried were placed into a Gulnar twin-screw extruder (Figure 1c). The extruder had a screw diameter of 25 mm and an L/D ratio of 42. To achieve the GO concentrations specified in Table 2, the masterbatch with a 2% weight ratio of GO was diluted and integrated into the extrusion process. The polymer nanocomposite formulation nomenclatures and the weight ratios of the GO are presented in Table 2.

The temperature gradient values were 210–215–220–215–220–225–230–235 °C. During the process, the screw speed was set to 100 rpm for all mixtures. During the extrusion process, a GO content of 2 wt.% was found to significantly reduce viscosity, limiting industrial processing; thus, samples were produced with a maximum of 1 wt.% GO content. Test specimens were obtained by using an Xplore MC 40 micro-compounder (Figure 1d) and an Xplore IM 12 micro-injection molder (Figure 1e). The mixing process was carried out at a screw speed of 100 rpm and a residence time of 2 min, with the barrel temperature sustained at 230 °C. Nanocomposites were transferred to the injection molding machine via a transfer cylinder and immediately subjected to molding. The barrel temperature and mold temperature of the micro-injection molding device were set to 230 °C and 80 °C, respectively, and the injection pressure was fixed at 10 bar. A schematic representation of the manufacturing process of the PA6/GO nanocomposite test specimens is shown in Figure 1.

### 2.3. Mechanical Tests

**Figure 2:**
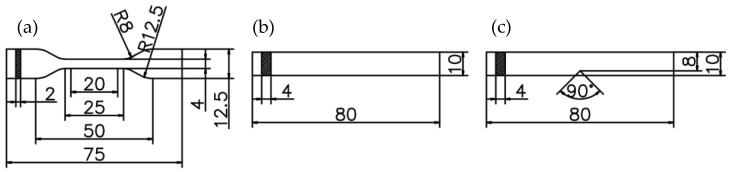
(**a**) ISO 527-2/5A tensile, (**b**) ISO 178 three-point bending, and (**c**) ISO 179 notched Charpy impact test specimen dimensions.

Mechanical evaluations were conducted, including tensile, bending, and impact testing. The experiments were performed in a laboratory maintained at a temperature of 25 ± 2 °C and a humidity of 50 ± 5%. Five samples were used for each group. Tensile, three-point bending, and notched Charpy impact test specimens of PA6 and PA6/GO were manufactured in accordance with the dimensions specified by ISO 527-2/5A [37], ISO 178 [38], and ISO 179 [39] standards, respectively. Figure 2 represents a scheme with the dimensions of all the specimens.

**Figure 3:**
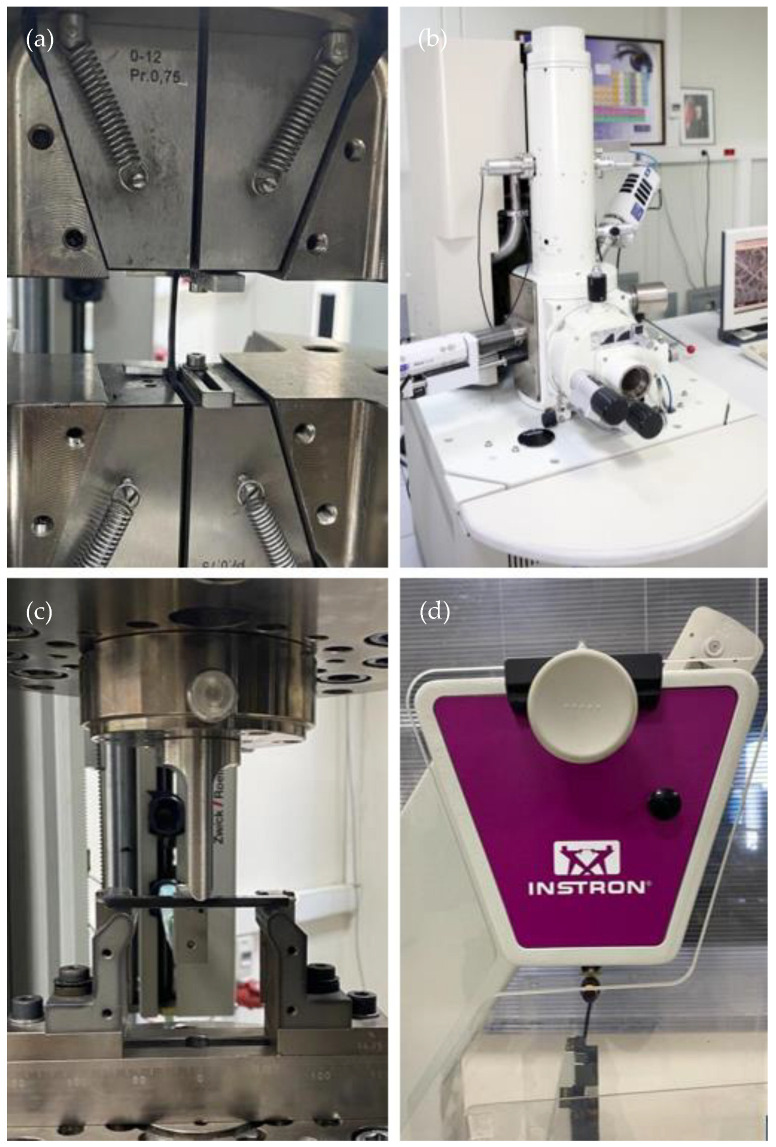
(**a**) Tensile test, (**b**) SEM, (**c**) three-point bending test, and (**d**) notched Charpy impact test.

Tensile tests were performed at three different temperatures: 25 °C, 50 °C, and 70 °C. As shown in Figure 3a, tensile tests were conducted using a ZwickRoell Z250 universal testing machine, Ulm, Germany. To determine the modulus of elasticity, the specimens were tested at a constant crosshead speed of 1 mm/min with the assistance of an extensometer. Following the determination of the modulus of elasticity, tensile tests were conducted at a constant crosshead speed of 5 mm/min. The fracture morphologies of the tensile test specimens performed at 25 °C were observed using JEOL/JSM-6510LV SEM, Tokyo, Japan (Figure 3b). Working distance and the voltage were 13 mm and 15 kV, respectively. Three-point bending tests were performed in accordance with ISO 178 standard using a ZwickRoell Z250 universal testing machine, Ulm, Germany (Figure 3c). To determine the bending modulus, the tip of the extensometer was placed at the center of the test specimens, and the experiments were conducted at a constant speed of 1 mm/min. Subsequent to ascertaining the flexural modulus, the three-point bending tests were conducted at 5 mm/min. The notched Charpy impact tests were performed in accordance with ISO 179 standard using an Instron Ceast 9050 Charpy impact tester, Massachusetts, USA (Figure 3d). Based on preliminary studies on PA6/GO specimens, the hammer impact energy was 5 J.

### 2.4. Fatigue

**Figure 4:**
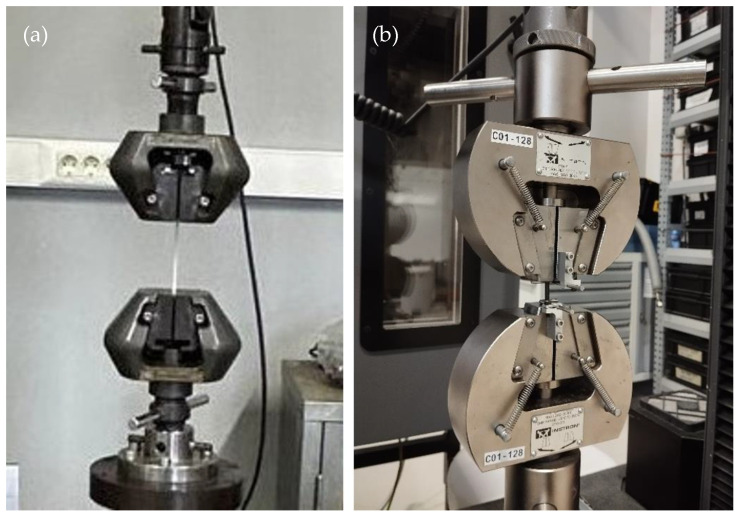
(**a**) Fatigue and (**b**) creep test specimen mounted between grips.

Fatigue tests were performed using a DARTEC 100 kN servo-hydraulic testing machine, Surrey, UK. The experiments were performed in a laboratory maintained at a temperature of 25 ± 2 °C and a humidity of 50 ± 5%. PA6 and PA6/GO fatigue specimens were prepared according to ISO 527-2/5A standard dimensions. The tests were conducted in a stress-based tension–tension load control mode at a frequency of 1 Hz with a stress ratio of 0.1. An extensometer was fixed to the gauge length of the specimen to measure elongation. The initial maximum stress level was determined to be approximately 60% of the UTS. Three specimens were tested at each stress level. Preliminary trials were conducted to determine the loading for the target cycle rates for each specimen. The tests were conducted up to 10^5^ cycles. The fatigue test specimen mounted between the grips is demonstrated in Figure 4a.

### 2.5. DMA

The Mettler Toledo dynamic mechanical analysis test device was used in a three-point bending configuration for the DMA tests that were carried out. The dimensions of the test specimen were 4 mm in length, 10 mm in width, and 35 mm in height. In order to determine the linear deformation zone of the specimens, a strain amplitude sweep test was carried out prior to the testing. PA6 and PA6/GO test specimens were subjected to a frequency of 1 Hz and a stress amplitude of 20 μm during the testing process. In the course of the DMA testing, the heating rate was established at 3 °C/min, and the viscoelastic characteristics of the PA6 and PA6/GO nanocomposites were examined in the temperature range of 25 °C to 150 °C.

### 2.6. Creep

At both 25 °C and 70 °C, creep tests were carried out with the assistance of an Instron 5982 universal testing machine, Norwood, MA, USA (Figure 4b). The PA6 and PA6/GO creep specimens were manufactured in accordance with the standard dimensions established by ISO 527-2/5A requirements. For each group, there were three specimens that were examined. When conducting the creep tests, the load that was equivalent to sixty percent of the yield stress of each group was increased at a rate of 1 mm/min. At the same time, the elongation against a constant load was simultaneously recorded for ten hours.

## 3. Results and Discussion

### 3.1. Mechanical Tests

**Figure 5:**
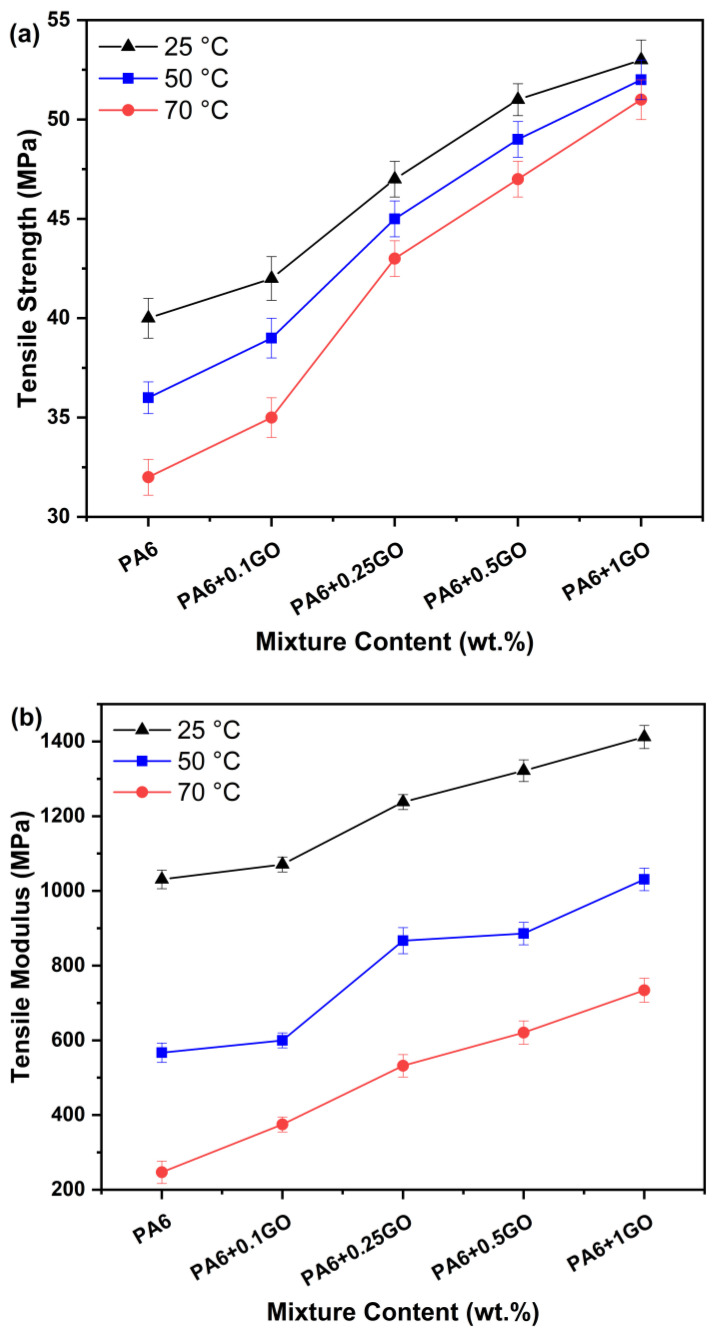
(**a**) Tensile strength and (**b**) elastic modulus of PA6 and PA6/GO nanocomposites at 25 °C, 50 °C, and 70 °C.

In Figure 5a,b, the results of tensile tests performed at three different temperatures, 25 °C, 50 °C, and 70 °C, on PA6 and PA6 nanocomposites are shown. Upon analyzing the results, it was noted that as GO increases, the maximum tensile strength and elasticity modulus of PA6/GO nanocomposites increase. However, when the temperature increased, the maximum tensile strength and elasticity modulus values decreased.

When Figure 5a,b were examined, it was seen that at 25 °C, the tensile strength and elastic modulus for PA6 were 40 MPa and 1031 Mpa, respectively. For PA6 + 0.25GO, the tensile strength and elastic modulus attained 47 Mpa and 1238 Mpa, reflecting enhancements of 11.9% and 15.59% relative to PA6 + 0.1GO. However, the increase did not persist in the same manner subsequently, and a decline in the rate of rise was observed. The tensile strength and modulus of elasticity for PA6 + 0.5GO were 51 Mpa and 1322 Mpa, showing increases of 8.51% and 6.78% relative to PA6 + 0.25GO. The tensile strength and elasticity modulus for PA6 + 1GO achieved 53 Mpa and 1412 Mpa, representing an increase of 3.102% and 6.81% compared to PA6 + 0.5GO, respectively.

The impact of changes in temperature on tensile strength, alongside the variation in GO content, was also examined. After raising the temperature from 25 °C to 50 °C, the tensile strength of PA6 diminished by 10%. Correspondingly, as the temperature increased from 50 °C to 70 °C, the tensile strength of PA6 decreased by 11.11%. However, the reduction in tensile strength occurred gradually with the increasing amount of GO. For PA6 + 1GO, increasing the temperature from 25 °C to 50 °C resulted in a reduction in tensile strength of 1.88%. Likewise, when the temperature was elevated from 50 °C to 70 °C, the reduction rate in tensile strength for the PA6 + 1GO sample decreased to 1.92%. While examining the elasticity modulus values at different temperatures, it was seen that with an increase in temperature, the elasticity modulus values diminished by over fifty percent. While a definitive correlation was not evident as observed with tensile strength, the enhancement of GO concentration mitigated the reduction in the elasticity modulus as temperature increased. Similar observations were made in studies involving GO-filled PA6 nanocomposites, where the incorporation of GO significantly improved the mechanical and thermal stability of the materials at elevated temperatures [40]. These findings are consistent with those presented by Jing et al. [41], who investigated the enhancement of mechanical and thermal properties in polyurethane and poly(ethylene chloride)-based nanocomposites with FGO. The authors reported a marked improvement in both mechanical strength and thermal stability upon the incorporation of FGO. Specifically, mechanical strength increased by 25%, and thermal stability improved by 30% with the addition of FGO.

As the temperature rises, a decrease in mechanical and thermal properties of PA6 is expected [35]; however, the addition of GO reduced this loss and enhanced the material’s strength, load-carrying capacity, and thermal stability. At lower temperatures, GO-filled nanocomposites showed more rigid and stiff behavior. As the temperature increases, the viscosity of PA6 decreases, and the material’s deformation capacity increases. However, in GO-filled nanocomposites, due to GO’s superior mechanical and thermal properties, the deformation of the PA6 matrix was limited, and the nanocomposites exhibited better structural integrity, becoming more resistant to temperature.

The addition of GO to the polymer matrix provided greater resistance under stress, thus increasing the tensile strength and elasticity modulus values. Additionally, GO’s contribution increased thermal conductivity, enhancing the material’s resistance to high temperatures and improving its thermal stability [42]. In a study conducted by Graziano et al. [43], after adding GO to HDPE nanocomposites through a combination of solvent and melt mixing methods, significant increases were observed in the elasticity modulus and maximum tensile strength in mechanical tests. Heydari et al. [44], in their study, demonstrated that GO nanoparticles led to significant improvements in the mechanical and thermal properties of polyurethane and polycaprolactone-based nanocomposites. The addition of GO increased the tensile and bending strength, raised the elastic modulus, and made the composites more resistant to temperature changes.

**Figure 6:**
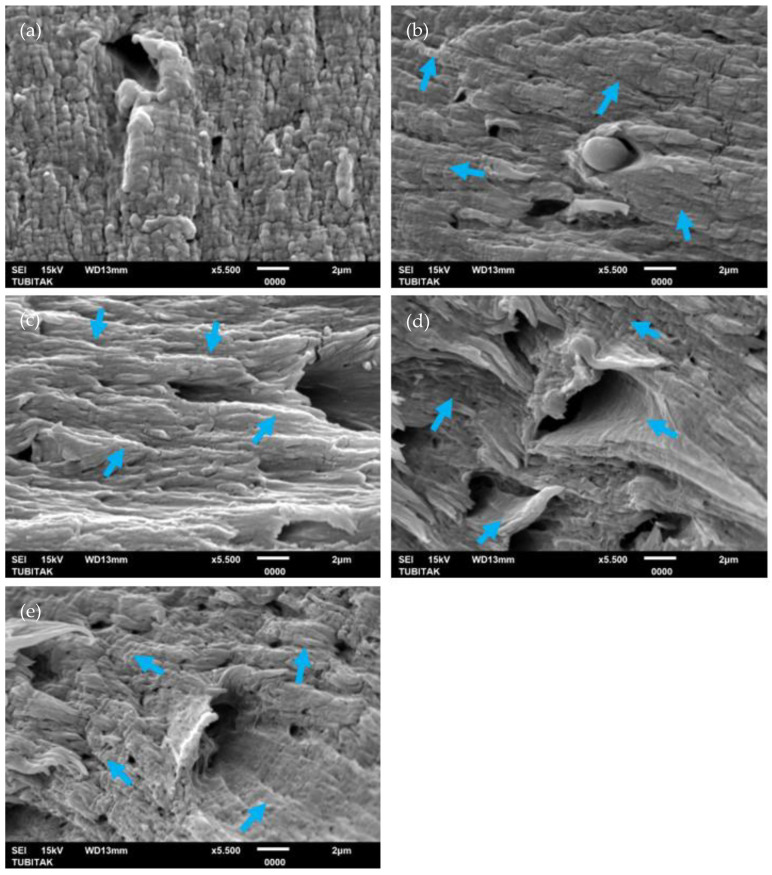
SEM micrographs of PA6 and PA6/GO nanocomposites: (**a**) PA6, (**b**) PA6 + 0.1GO, (**c**) PA6 + 0.25GO, (**d**) PA6 + 0.5GO, and (**e**) PA6 + 1GO. Arrows indicate river markings.

The morphology of the fracture surfaces of PA6 and PA6/GO nanocomposite test samples post-tensile testing is depicted in Figure 6a–e. These images clearly demonstrate the gradually increased rigid fracture nature of nanocomposites while increasing the GO content. This rigid behavior explains the increase in tensile strength and elasticity modulus properties in GO-filled nanocomposites. One of the fundamental issues typically encountered in nano-filled materials is agglomeration. As noted by Gudarzi et al. [45], achieving optimal dispersion and ensuring strong interfacial bonding are critical for maximizing the material’s performance in various applications. Sun et al. [46] also noted considerable enhancements in the tensile strength, modulus, and thermal stability of PA6 composites upon the incorporation of GO.

The river markings noted on the right side of Figure 6e, especially in the PA6 + 1GO, indicate a more significant effect potentially linked to the aggregation of GO nanosheets. The rigid structure of the GO particles facilitates the initiation of energy absorption processes alongside crack propagation. Despite the absence of visual identification of GO in the SEM micrographs of Figure 6b–e, the apparent structural alterations, including surface roughness and undulations indicated by arrows, suggest that the incorporation of GO substantially modified the fracture morphology of the PA6 matrix. The lack of visible GO agglomerates, combined with surface roughness and undulations, indicates a relatively efficient dispersion of GO inside the PA6 matrix [47]. Thanks to the SEM, the use of a combination of solvent mixing with melt mixing can be identified as a strong alternative for the agglomeration problem and supports the separation and distribution of GO layers through facilitating the manufacturing process [48].

**Figure 7:**
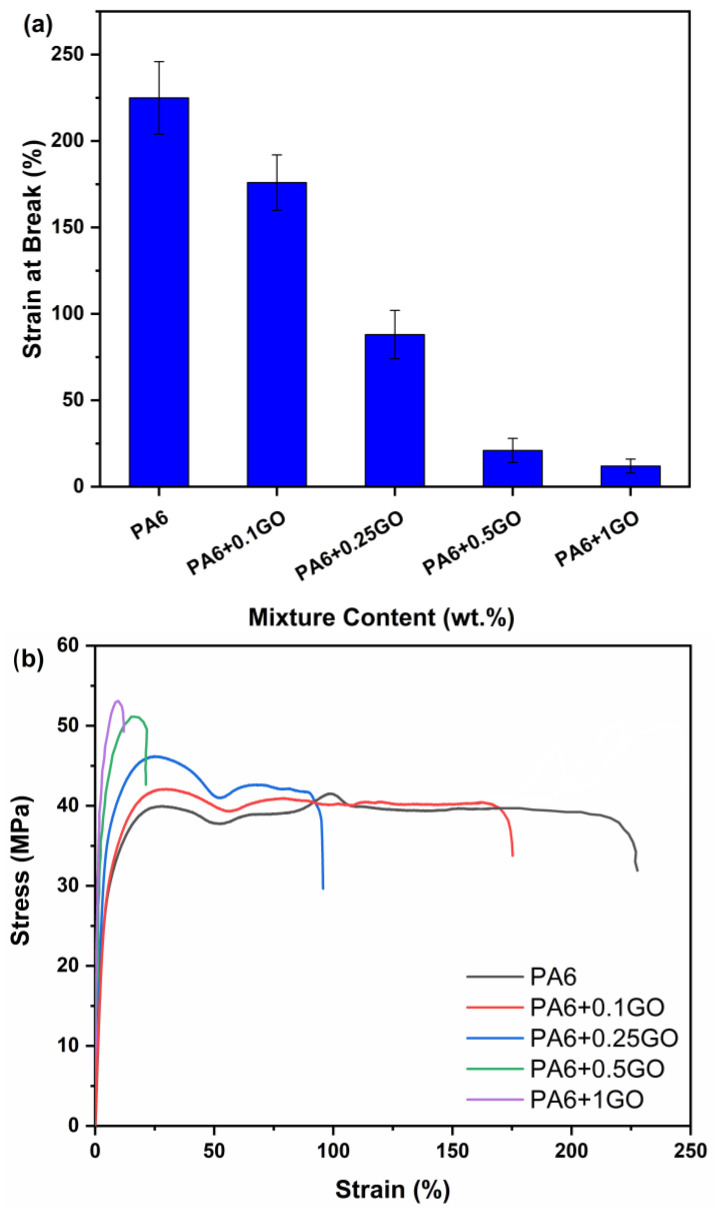
(**a**) Strain at break and (**b**) stress–strain curves for PA6 and PA6/GO nanocomposites.

Figure 7a,b shows the strain at break values and stress–strain curves for PA6 and PA6/GO nanocomposites. When evaluating strain at break and SEM images together, it is evident that the increase in GO content has reduced strain at break. The decrease in strain at break becomes clearly observable starting from the PA6 + 0.1GO nanocomposite. The rigid structure of GO increased the rigidity of the nanocomposites, limited the deformation of PA6/GO nanocomposites, and resulted in a decrease capacity to deform [49,50,51].

**Figure 8:**
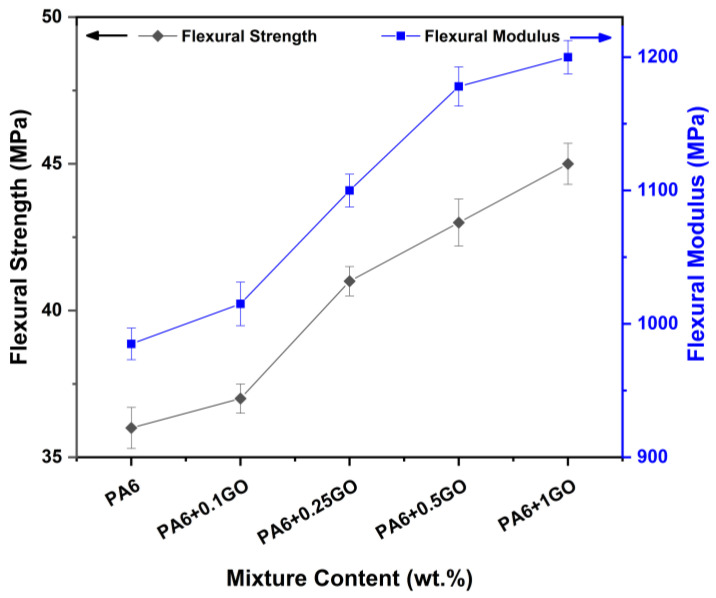
Flexural properties of PA6 and PA6/GO nanocomposites.

Figure 8 demonstrates the bending stress and bending modulus data for PA6 and PA6/GO nanocomposites. The rigidity property of GO affected the PA6/GO nanocomposites, showing that with the increase in GO content, the bending stress and bending modulus significantly increased. This increase follows a similar trend to the tensile test data, continuing up to PA6 + 0.25GO, after which the rate of increase decreases. Upon examining the graph, it was observed that the bending stress and bending modulus values of the PA6 were 36 MPa and 985 MPa, respectively. The bending stress and bending modulus of PA6 + 0.1GO increased by 2.78% and 3.05%, reaching 37 MPa and 1015 MPa; the bending stress and bending modulus of PA6 + 0.25GO reached 41 MPa and 1100 MPa, showing an increase of 10.81% and 8.37% compared to PA6 + 0.1GO. However, from PA6 + 0.5GO onwards, the rate of increase decreased, and the bending stress and bending modulus reached 43 MPa and 1178 MPa, showing an increase of 4.88% and 7.09% compared to PA6 + 0.25GO. Similarly, the bending stress and bending modulus of PA6 + 1GO reached 45 MPa and 1200 MPa, showing an increase of 4.65% and 1.87% compared to PA6 + 0.5GO.

**Figure 9:**
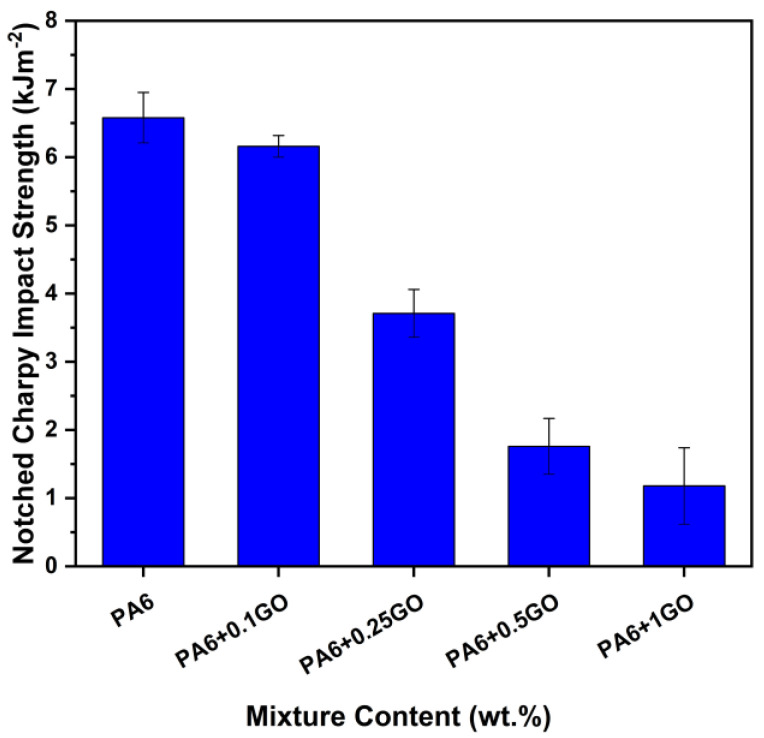
Notched Charpy impact properties of PA6 and PA6/ GO nanocomposites.

Figure 9 illustrates that the influence of GO’s mechanical characteristics on the notched Charpy impact test outcomes was adverse. The integration of GO led to a decrease in impact strength by obstructing the stress transfer pathway in the nanocomposite samples, hence promoting crack development. Consequently, it was noted that the impact strength was less in all samples containing GO in comparison to PA6. As the concentration of GO content rose, the energy absorption capacity of the resultant nanocomposite samples diminished, resulting in a decline in the impact strength property of the nanocomposites [52]. The findings on elongation at break were consistent. The impact strength values are as follows: 6.58 kJ/m^2^ for PA6, 6.18 kJ/m^2^ for PA6 + 0.1GO, 3.81 kJ/m^2^ for PA6 + 0.25GO, 1.76 kJ/m^2^ for PA6 + 0.5GO, and 1.18 kJ/m^2^ for PA6 + 1GO. As noted by Gudarzi et al. [45], increased rigidity and reduced impact strength should be considered.

### 3.2. Fatigue

**Figure 10:**
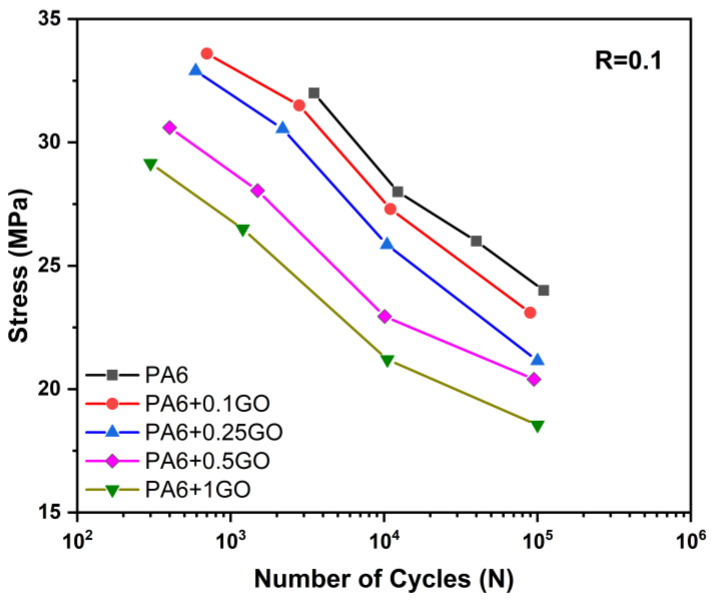
S-N curves of PA6 and PA6/GO nanocomposites.

Figure 10 shows the fatigue strength of PA6 and PA6/GO nanocomposites subjected to tensile stress. The fatigue strength of the samples was determined by loading them with different amounts of tensile strength values obtained from tensile tests.

Ramkumar et al. [53] indicated that PA6 nanocomposites, particularly those reinforced with CNTs, exhibit significantly higher fatigue resistance compared to pure PA6. The nanocomposites demonstrated longer fatigue life, with crack initiation occurring at later stages due to the strengthening of the microstructure. Furthermore, the incorporation of nanomaterials enhanced the structural integrity of PA6. For instance, while the fatigue life of pure PA6 was approximately 10^4^ cycles, CNT-reinforced PA6 nanocomposites achieved a fatigue life of up to 10^6^ cycles. However, as seen in the elongation at break and impact tests, the rigid GO particles hindered the deformation capability of the matrix and facilitated crack propagation. As a result, the load-carrying capacity of the nanocomposites decreased with increasing GO content in all cycles. While the PA6 reached 10^5^ cycles at 60% of its tensile strength, PA6 + 1GO was able to reach 10^5^ cycles at 35% of its tensile strength.

**Figure 11:**
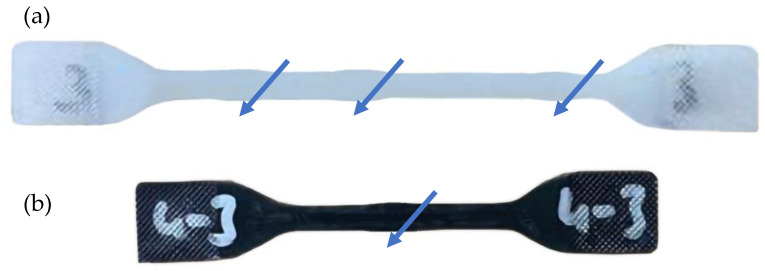
(**a**) PA6 and (**b**) PA6 + 1GO test specimens after 10^5^ cycles. Arrows indicate necking.

At all stress levels, the deviations in PA6/GO nanocomposites were seen to be less than those in PA6. Since this deviation can primarily be attributed to creep, it can be said that PA6/GO samples demonstrate better creep resistance compared to PA6. As shown in Figure 11, the PA6 polymer exhibited necking behavior at multiple points due to local neck formation and thermal softening during the tests. However, PA6/GO nanocomposite samples did not exhibit such a phenomenon. The addition of GO to the PA6 was thought to have reduced the unit elongation of the PA6 matrix and increased the elasticity modulus, thereby reducing the hysteresis heating caused by viscous effects [53].

When the fatigue curves are examined, three different regimes are observed depending on the applied stress amplitudes. The fatigue curves exhibited a rapid regime (I) during the initial cycles, followed by a gradual regime (II), and a sharp decline before failure (III). Compared to PA6, it can be said that PA6/GO nanocomposites exhibit an increase in damage kinetics. This situation could be due to the presence of weak polymer-continuous reinforcement interfaces in the samples [54].

### 3.3. DMA

**Figure 12:**
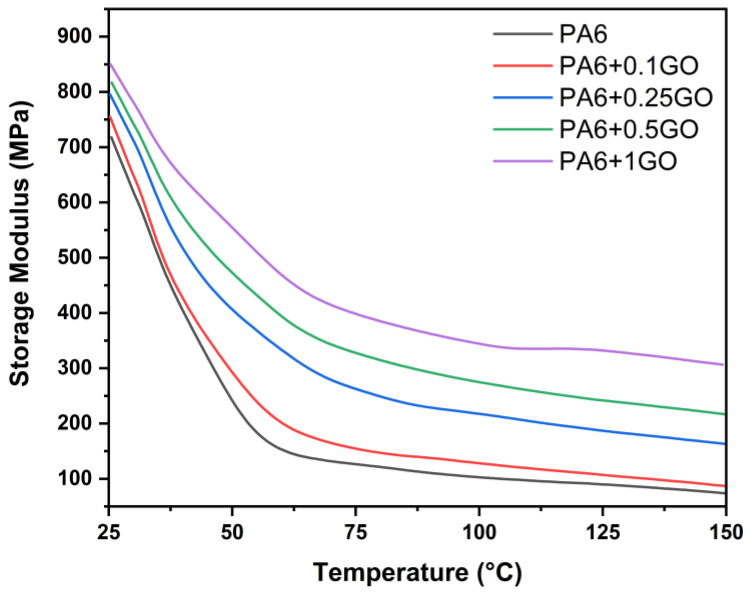
Storage modulus results of PA6 and PA6/GO nanocomposites.

**Figure 13:**
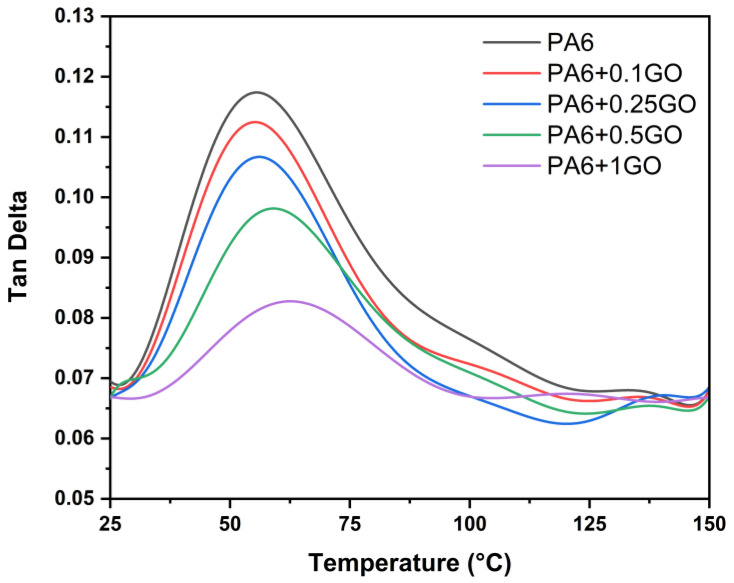
Tan Delta results of PA6 and PA6/GO nanocomposites.

The storage modulus and Tan Delta graphs for PA6 and PA6/GO nanocomposites are examined as a function of temperature at a constant frequency of 1 Hz in Figure 12 and Figure 13. The impact of temperature is seen in Figure 12’s storage modulus curve. The storage modulus values steadily dropped as the temperature rose. Furthermore, compared to PA6, the storage modulus of PA6/GO samples increased at all temperature ranges as the GO content increased. The stiffness brought about by the addition of GO to the PA6 matrix explains this rise in the nanocomposites’ storage modulus. Zhao et al. [55] indicated that the results revealed a significant increase in the storage modulus with the addition of GNP, indicating enhanced stiffness of the composites. Stiffness, strength, and storage modulus have all improved as a result of the increased GO content; these findings are corroborated by the elasticity modulus values found in the tensile testing [56,57,58].

For PA6 and PA6/GO nanocomposites, the Tan Delta, a measure of the damping, increased to the T_g_, as shown in Figure 13. It then exhibited a declining tendency until it reached the stress relaxation area after attaining T_g_. By limiting matrix deformation through the development of tiny nucleation, the stiff GO distributed throughout the PA6 matrix has demonstrated its impact on the damping coefficient of PA6/GO nanocomposites. The drop in Tan Delta values and the rise in the T_g_ indicated how effective the addition was in the polymer. Accordingly, a decrease in the maximum Tan Delta value was seen when looking at Figure 13. Nevertheless, T_g_ rose because of the stiff GO addition and its uniform dispersion throughout the PA6/GO matrix. The values on the storage modulus graph also supported the observation. These outcomes demonstrated the effectiveness of resolving the agglomeration issue and reaching the desired homogenous distribution by combining melt and solvent mixing techniques. They were made possible by the homogeneous distribution of GO in the PA6 matrix.

### 3.4. Creep

**Figure 14:**
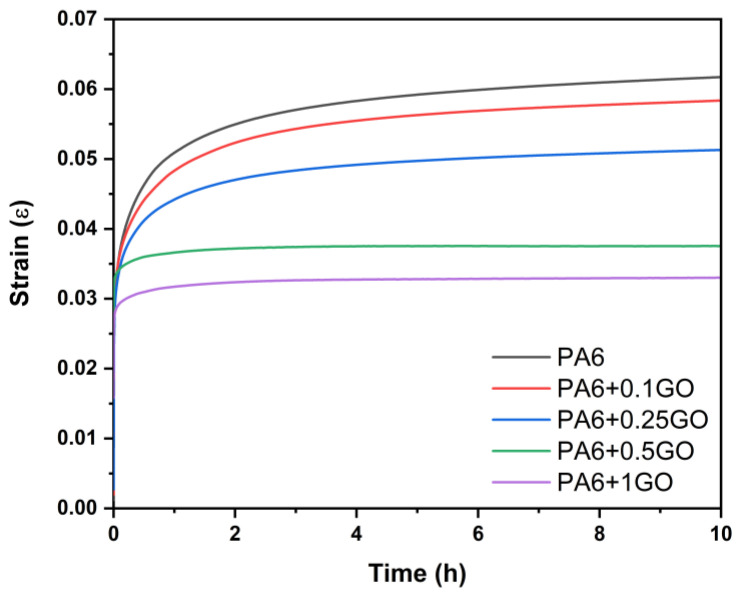
Creep properties of PA6 and PA6/GO nanocomposites at 25 °C.

As shown in Figure 14, the creep strain of PA6/GO nanocomposites is lower than that of PA6, which is expected as the rigid GO increases creep resistance [59,60,61]. The increase in creep resistance of nanocomposites containing stiff GO is due to the decrease in both the viscoelastic and viscoplastic components of the overall creep strain, in addition to the increasing modulus of elasticity with GO content. Rigid GO restricted the PA6 matrix, thereby limiting the viscous flow of the polymer, and consequently increased the creep resistance. Homogeneous distribution of GO enhanced load transfer and ensured that PA6/GO nanocomposites exhibited better creep resistance [60,61]. After a 10 h creep test, the decrease in creep strain was observed as follows: 6.5% for PA6/GO nanocomposites containing 0.1 wt.% GO, 17.8% for 0.25 wt.% GO, 39.5% for 0.5 wt.% GO, and 52.4% for 1 wt.% GO. This result demonstrates that rigid GO helps prevent long-term deformation by limiting the movement of polymer chains. The dispersion of rigid GO within the polymer matrix strengthens the mechanical properties of the matrix, making it more resistant to deformation, and increases the creep resistance in the viscoelastic and viscoplastic regions of the material. Furthermore, the tests showed that the creep modulus of the nanocomposites increased with GO reinforcement. The creep modulus is directly related to the material’s resistance to deformation, and the results indicate that GO-containing nanocomposites demonstrated higher resistance to deformation under applied load. This can be explained by the interaction of GO with the polymer matrix, which makes the load distribution more homogeneous, thus creating a more durable structure. Bakbak et al. [62] investigated the creep test results, revealing that the addition of G significantly improved the creep resistance of epoxy-nanocomposites. The incorporation of G into the epoxy matrix reduces the creep strain over time, with the nanocomposite showing lower deformation rates compared to pure epoxy. Specifically, the epoxy/graphene nanocomposite exhibits a notable reduction in the steady-state creep rate, with a reduction of approximately 30% compared to pure epoxy. This improvement is attributed to the strong interfacial bonding between the graphene sheets and the epoxy matrix, which restricts the mobility of the polymer chains, thereby mitigating creep deformation.

**Figure 15:**
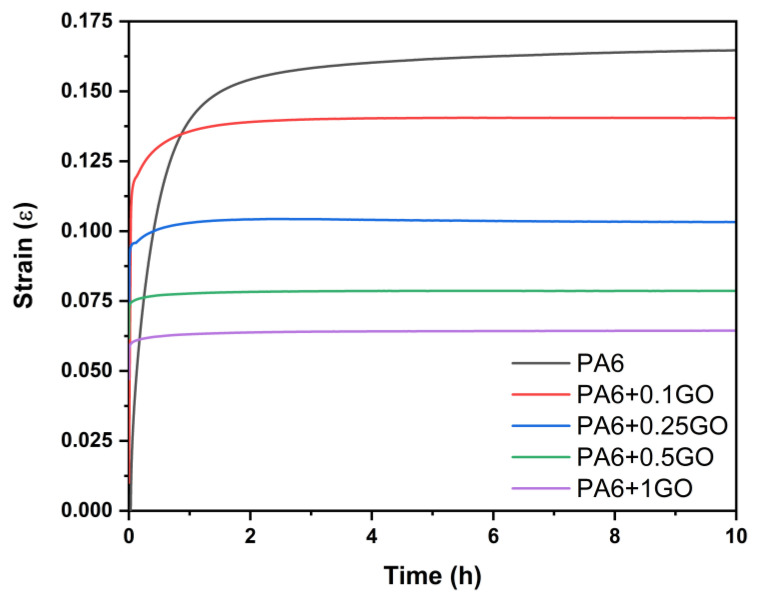
Creep properties of PA6 and PA6/GO nanocomposites at 70 °C.

Figure 15 shows the creep test results conducted at 70 °C. This investigation, performed at a temperature of 20 °C above the T_g_ of roughly 50 °C, as illustrated in Figure 13, revealed that the integration of GO in nanocomposites resulted in less creep deformation compared to PA6 at elevated temperatures, while also preserving better structural integrity. When evaluated together with the storage modulus results, compared to PA6, the storage modulus of PA6/GO samples increased at all temperature ranges as the GO content increased. The thermal conductivity of GO allowed the nanocomposites to exhibit better resistance to high temperatures, which suggests that GO could be beneficial in applications where temperature fluctuations are significant. This result also aligns with the findings from tensile tests conducted at different temperature values. Wang et al. [63] investigated the creep behavior of CRGO-filled polystyrene (PS) composites at different temperatures. The creep tests demonstrated that the addition of CRGO to the polymer matrix enhanced creep resistance and reduced creep strain. At 70 °C, pure PS exhibited a creep strain of 0.5%, while the PS composite with 2 wt% CRGO showed a reduced creep strain of 0.3%.

## 4. Conclusions

In this study, the effective transfer of the superior properties of GO into nanocomposites and the investigation of their effects were the primary objectives. Mechanical, fatigue, and creep tests were conducted to assess the effect of GO on the polymer matrix.

- It was observed that increasing GO content improved the tensile and bending strengths of the PA6/GO nanocomposites. Tensile tests performed at different temperatures revealed that PA6’s tensile strength decreased with rising temperature, but the reduction in tensile strength in PA6/GO nanocomposites was less pronounced due to the reinforcing effect of GO. Charpy impact tests indicated that GO restricted strain, facilitating crack propagation and weakening the impact strength of the nanocomposites.
- Rigid GO reduced the cyclic strain amplitude of the PA6 matrix in PA6/GO nanocomposites and increased the modulus of elasticity, thereby reducing hysteresis heating caused by viscous effects. In contrast to PA6, which exhibited necking behavior at multiple points due to local neck formation and thermal softening, the PA6/GO nanocomposites did not show such behavior because of rigid GO.
- DMA results demonstrated that the storage modulus of PA6/GO nanocomposites increased with the addition of GO. Tan Delta values of nanocomposites decreased, and the Tg also rose, confirming the efficient dispersion and contribution of GO to the matrix.
- Creep tests revealed that PA6/GO nanocomposites had lower creep rates than PA6, indicating enhanced dimensional stability under stress.

## Figures and Tables

**Table 1:** Material properties of PA6 and GO.

PA6		GO	
Density	1.14 g/cm^3^	Bulk density	0.2–0.3 g/cm^3^
Viscosity number	145 mL/g	C:O atomic ratio	1.5
Melt volume rate	165 cm^3^/10 min	Layer numbers	3–10
Melting point	221 °C	Specific surface area	120–140 m^2^/g
VICAT softening	200 °C		
Tensile modulus	1000 MPa		
Yield strength	40 MPa		
Flexural modulus	900 MPa		
Flexural strength	35 MPa		
Charpy notched	4.5 kJ/m^2^		
Hardness Rockwell	120 Scale R		

**Table 2:** Mixtures of polymer nanocomposites.

Formulation	GO (wt.%)
PA6	0
PA6 + 0.1GO	0.1
PA6 + 0.25GO	0.25
PA6 + 0.5GO	0.5
PA6 + 1GO	1

## Data Availability

The article includes the necessary data; further inquiries can be directed to the corresponding author.

## Abbreviations

The following abbreviations are used in this manuscript:

AFM	Atomic Force Microscope
CNTs	Carbon Nanotubes
DMA	Dynamic Mechanical Analysis
DSC	Differential Scanning Calorimetry
EVA	Ethylene-Vinyl Acetate
G	Graphene
GO	Graphene Oxide
HDPE	High-Density Polyethylene
Nano-SiO_2_	Nano-Silica
PA6	Polyamide 6
rGO	Reduced Graphene Oxide
SEM	Scanning Electron Microscope
SiC	Silicon Carbide
SiO_2_	Silicon Dioxide
T_g_	Glass Transition Temperature
TGA	Thermogravimetric Analysis
